# Corneal Clarity and Visual Outcomes after Small-Incision Lenticule Extraction and Comparison to Femtosecond Laser-Assisted In Situ Keratomileusis

**DOI:** 10.1155/2017/5646390

**Published:** 2017-03-15

**Authors:** Apostolos Lazaridis, Konstantinos Droutsas, Walter Sekundo, Michael Petrak, Stephan Schulze

**Affiliations:** Department of Ophthalmology, Philipps University of Marburg, Marburg, Germany

## Abstract

*Purpose*. To evaluate corneal clarity and visual outcomes after small-incision lenticule extraction (SMILE) and compare them to femtosecond laser-assisted in situ keratomileusis (FS-LASIK). *Materials and Methods*. Fifty-eight myopic eyes of 33 patients who underwent SMILE were compared to 58 eyes of 33 patients treated with FS-LASIK. All procedures were performed using VisuMax® femtosecond laser and MEL 80® excimer laser (Carl Zeiss Meditec AG, Germany). Pentacam™ (Oculus, Germany) was used for pre- and 3-month postoperative corneal densitometry (CD) analysis. CD was evaluated at 3 optically relevant, concentric radial zones (0–2 mm, 2–6 mm, and 0–6 mm annulus) around the corneal apex and at 3 different anatomical corneal layers (anterior, central, and posterior). Associations of postoperative CD values with the lenticule thickness and ablation depth were examined. Preoperative and postoperative corrected distance visual acuity (CDVA) values were also compared. *Results*. After SMILE, the total CD (all corneal layers) at 0–6 mm annulus showed no significant change compared to preoperative values (*P* = 0.259). After FS-LASIK, the total CD was significantly reduced (*P* = 0.033). Three-month postoperative CD showed no significant differences between the 2 groups for all examined annuli (0–2 mm: *P* = 0.569; 2–6 mm: *P* = 0.055; and 0–6 mm: *P* = 0.686). Total CD after SMILE at 0–6 mm annulus displayed a weak negative association with the lenticule thickness (*P* = 0.079, *R*^2^ = 0.0532) and after FS-LASIK displayed a weak negative association with the ablation depth (*P* = 0.731, *R*^2^ = 0.0015). Postoperative CDVA was similar for both groups (*P* = 0.517). *Conclusion*. Quantification of corneal clarity using the Scheimpflug CD showed similar results before and 3 months after SMILE. Compared to FS-LASIK, no significant differences of corneal clarity and CDVA were found 3 months postoperatively.

## 1. Introduction

The main issues of concern for refractive surgeons and patients undergoing corneal refractive surgery are the predictability and long-term stability of attempted correction, the quality of the visual outcome, and most importantly the safety of the procedure, in regard to long-term corneal biomechanical stability and minimization of intra- and postoperative complications. According to recent published studies [1–5], the small-incision lenticule extraction (SMILE) technique seems to provide equal or better results regarding these issues compared to femtosecond laser-assisted in situ keratomileusis (FS-LASIK). From the patients' perspective, however, the fast visual recovery is also an important factor along with precision of the correction, long-term stability, and safety.

SMILE procedure minimizes epithelial injury and release of cytokines and growth factors from the wounded epithelium [6]. Moreover, the early postoperative inflammatory and wound healing response has been reported to be less reactive after an all-femtosecond laser-assisted procedure compared to FS-LASIK [6–8]. However, a transient haze-like reaction can be clinically observed at a slit-lamp examination after SMILE procedure. Thus, the visual rehabilitation might be prolonged compared to FS-LASIK. The corneal opacification usually regresses and becomes clinically insignificant within the first year.

This effect has been quantified in a previous study by means of in vivo confocal microscopy [9]. After comparing corneal backscatter from anterior stroma between SMILE and FS-LASIK, the authors concluded that SMILE results in statistically significant increase of backscattered light intensity in the anterior stroma at 1-week, 1-month, and 3-month postoperative examinations.

Another method of objective assessment of corneal clarity is provided by the Scheimpflug corneal densitometry (CD), which is a noninvasive method of quantifying corneal opacification by detecting backscattered light [10]. This method has been used for the evaluation of regression or progression of various corneal pathologies such as infectious keratitis [11], corneal dystrophies [12], and keratoconus [13] and allowed objective monitoring of corneal clarity following keratoplasty [14–16], corneal collagen crosslinking [17–19], and corneal refractive surgery [20, 21].

In the present study, we evaluated the preoperative and 3-month postoperative corneal clarity in a group of eyes which underwent SMILE surgery for myopia and astigmatism by measuring the amount of backscattered light from the different regions of the cornea using the CD software of Pentacam (Oculus, Germany). The same measurements were conducted for a group of eyes which underwent FS-LASIK. We compared the results within each group (preoperative versus 3-month postoperative values) and also compared the CD status between the 2 groups before and after surgery. Possible associations between postoperative CD values and the lenticule thickness for SMILE group or the ablation depth for FS-LASIK group were examined. Finally, the preoperative and postoperative corrected distance visual acuities (CDVA) were compared within the two groups and between them.

## 2. Materials and Methods

### 2.1. Subjects

In our retrospective comparative case series, the data were obtained from 58 myopic eyes of 33 patients who underwent SMILE. This group was compared to an equal number of FS-LASIK-treated eyes (*n* = 58) of 33 patients. Each SMILE patient was treated within the same session on the same surgical day with a FS-LASIK patient during the study period. All surgeries were performed by the same surgeon (WS). The procedures were performed using the VisuMax platform consisting of VisuMax femtosecond laser and MEL 80 excimer laser (Carl Zeiss Meditec AG, Germany).

All patients underwent preoperatively an ophthalmic evaluation that included autorefraction, pupillometry, uncorrected distance visual acuity (UDVA), corrected distance visual acuity (CDVA), manifest and cycloplegic refraction, slit-lamp examination of the cornea, the anterior segment and the retina, tear film testing (tear production evaluated with Schirmer's test II, and tear evaporation evaluated with tear break up time test), IOLMaster® 500 (Carl Zeiss Meditec AG, Germany) biometry, and finally corneal tomography and CD analysis using Pentacam. The measurements were repeated 3 months postoperatively with the exception of IOLMaster biometry and cycloplegic refraction.

The inclusion criteria for the patients that participated in the study groups were the stable refraction 2 years prior to surgery and a normal preoperative corneal tomography. The range of the refractive error and refractive correction was not part of our inclusion criteria. Exclusion criteria were any optical opacities or pathology on slit lamp, previous corneal surgeries, ocular trauma or intraocular surgery, severe dry eye, corneal disease or ocular infection, and collagen vascular/autoimmune diseases.

### 2.2. Femtosecond Laser-Assisted In Situ Keratomileusis Procedure

FS-LASIK was performed after application of topical anaesthesia. Flaps were cut using the VisuMax femtosecond laser with energy settings at 180 nJ, repetition rate at 500 kHz, pulse duration between 220 and 580 femtoseconds, and laser spot separation of 5 *μ*m for lamellar flap cut and 2 *μ*m for the flap side cut. During the docking process, the patient was asked to observe the green blinking fixation light under dim surrounding illumination and the suction was initiated. After both flaps (the right eye followed by the left eye) were precut, the patient was rotated on the same bed under the MEL 80 excimer laser. Here, the periocular area was draped with sterile plastic foil and a suction speculum connected to a pump was inserted. The surface of the cornea was flushed with BSS. The flap was lifted using 2 blunt spatulas, and the ablation started. The flap was repositioned, and the interface was flushed with BSS. The flap surface was stretched with sponges and care was taken to watch the uniformity of the gutter in order to avoid flap striae. After the speculum was removed, antibiotic and steroid drops were applied, the patient rotated back under VisuMax laser, and the flaps were examined using a build-in slit lamp of the femtosecond laser.

### 2.3. Small-Incision Lenticule Extraction Procedure

The energy was set at 160 nJ with a spot/track spacing of 4.5 *μ*m for the horizontal plane and 2 *μ*m for the side cut. An entering incision between 2.5 and 4 mm, depending on location and orbital features, was precut by the laser. The lenticule side cut thickness was set to 15 *μ*m. After entering the incision, the upper plane between the cap and the lenticule was separated first using the Chansue spatula followed by the separation of the lower refractive cut. Thereafter, the lenticule was removed using a modified serrated McPherson forceps. The pocket was flushed with BSS, and the interface was checked using the build-in slit lamp. In general, the entire length of the SMILE procedure was about half of the FS-LASIK surgery.

### 2.4. Corneal Densitometry Analysis

In our study, the corneal transparency was quantified using the Scheimpflug CD software, which is provided as an add-on to the standard software of Pentacam. With this noninvasive method, we can detect the amount of backscattered light in the different regions of the cornea. The densitometry data are recorded at four concentric radial zones (0–2 mm, 2–6 mm, 6–10 mm, and 10–12 mm annulus) around corneal apex as well as at three different anatomical layers of the cornea (anterior, central, and posterior). The anterior layer corresponds to the anterior 120 *μ*m, and the posterior layer corresponds to the most posterior 60 *μ*m of the cornea. The thickness of central corneal layer is defined by subtraction of the anterior and posterior layers from the total thickness. The total CD represents the backscatter rising from all corneal layers (i.e., reaching from epithelium to endothelium). The results are expressed in grayscale units (GSU). The GSU scale is calibrated by proprietary software, which defines a minimum light scatter of 0 (maximum transparency) and maximum light scatter of 100 (minimum transparency) [10]. Due to the fact that the white to white (WTW) diameter, as measured with IOLMaster 500, ranged from 11.4 to 12.9 mm in SMILE group and 11.8 to 12.8 mm in FS-LASIK group, we excluded the data from the 10–12 mm zone, since in cases of WTW diameter smaller than 12 mm, portions of the limbus and sclera would be included in the densitometry measurement of the outermost zone resulting in higher results. Moreover, we focused on the optically relevant 0 to 6 mm annulus (3 mm radius around corneal apex), since any opacification within this annulus would most likely affect the visual rehabilitation. Finally, the postoperative densitometry values of each patient were correlated with the thickness of the extracted lenticule or the depth of the photoablation.

### 2.5. Statistical Analysis

Descriptive statistics (i.e., mean, standard deviation, and range) were performed using the Microsoft Excel 2010 (Microsoft Corporation, Redmond, WA). Statistical analysis was performed using the SPSS version 20.0 (SPSS Inc., Chicago, IL). The normality of the distribution of all patient, surgical, CD, refractive, and visual data was evaluated with Shapiro–Wilk test. Wilcoxon signed-rank test for paired samples and paired sample *t*-test were used for nonparametric and parametric tests within each group. Mann-Whitney *U* test and independent sample *t*-test were used for nonparametric and parametric tests between the two groups. Linear regression analysis was performed in order to examine any possible relationship between the lenticule thickness or ablation depth (independent variables) and the postoperative CD values (dependent variable). All parametric and nonparametric tests were performed at the level of significance of 95% (0.05).

## 3. Results

### 3.1. Demographics and Variables

Mean values, standard deviation, and range of patients' demographics, refractive data, laser setting data, and white to white measurements are presented in Table 1. The preoperative and 3-month postoperative CD values for SMILE group are presented in Table 2 and for FS-LASIK group are presented in Table 3. The preoperative and 3-month postoperative refractive data and CDVA for both groups are presented in Table 4. The time frame between preoperative and postoperative examinations was 87 ± 5 days (range 76 to 98) for SMILE group and 89 ± 4 days (range 79 to 96) for FS-LASIK group.

### 3.2. Corneal Densitometry Analysis

**Figure 1:**
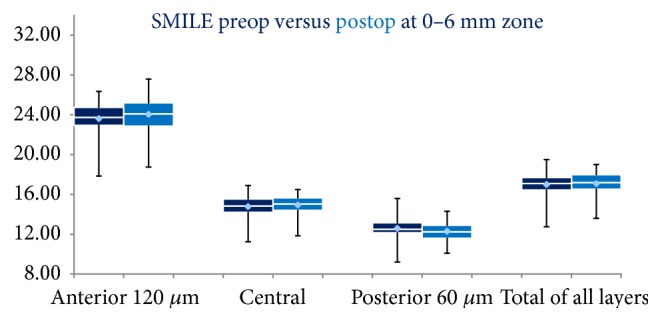
Box plots SMILE group. Preoperative densitometry values are presented with dark blue. Postoperative densitometry values are presented with light blue. Mean, median, maximum, and minimum values are being depicted.

At the 3-month postoperative examination, none of the patients included in our study demonstrated dry eye-related symptoms due to disturbance of tear film dynamics that would interfere with the results of CD measurements and CDVA. At 3 months after SMILE, the CD of the anterior 120 *μ*m at 0–6 mm annulus increased compared to preoperative status (*P* = 0.003). The postoperative CD of the central corneal layers was similar to preoperative values (*P* = 0.078). Interestingly, the postoperative CD of the posterior 60 *μ*m was reduced at statistically significant level (*P* = 0.012). The overall CD of all corneal layers (i.e., from epithelium to endothelium) at 0–6 mm annulus showed no significant change compared to preoperative values (*P* = 0.259) (Figure 1, Table 2).

**Figure 2:**
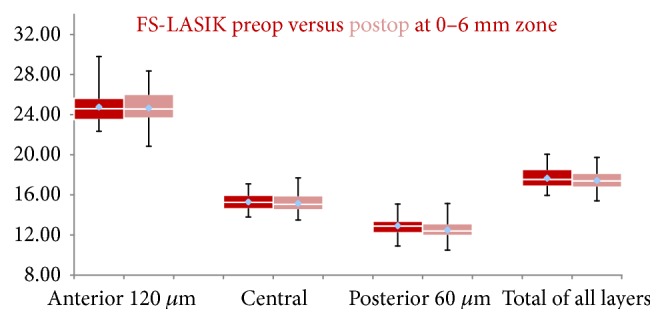
Box plots FS-LASIK group. Preoperative densitometry values are presented with red. Postoperative densitometry values are presented with pink. Mean, median, maximum, and minimum values are being depicted.

In FS-LASIK group, the postoperative CD of the anterior 120 *μ*m at 0–6 mm annulus was similar compared to preoperative status (*P* = 0.815). The CD of the central corneal layers was slightly reduced compared to preoperative values, without, however, reaching statistical significance (*P* = 0.059). Similarly to SMILE group, the postoperative CD of the posterior 60 *μ*m was reduced at statistically significant level (*P* = 0.001). The overall CD of all layers at 0–6 mm annulus showed a slight but yet statistically significant reduction compared to preoperative values (*P* = 0.033) (Figure 2, Table 3).

Comparing the total CD (all corneal layers) between the two groups, we concluded that there were no statistically significant differences in any of the examined annuli (*P* = 0.569 for 0–2 mm; *P* = 0.055 for 2–6 mm; and *P* = 0.686 for 0–6 mm annulus) at 3 months postoperatively (Table 5).

**Figure 3:**
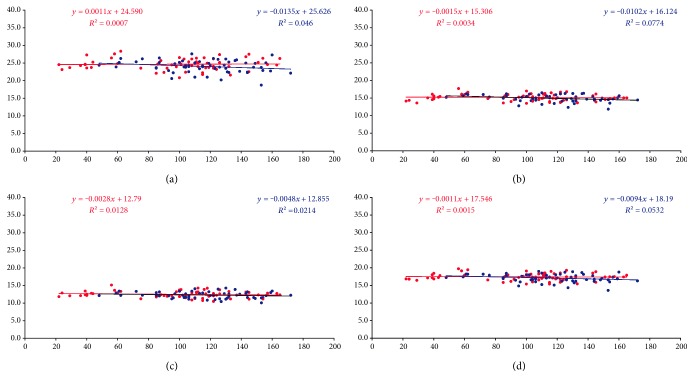
Linear regression analysis—scatterplots of the postoperative densitometry (*y*-axis —GSU units) of the anterior 120 *μ*m (a), central (b), posterior 60 *μ*m (c), and all layers of the cornea (d) at 0–6 mm annulus in relation to lenticule thickness (blue dots) versus ablation depth (red dots) (*x*-axis—*μ*m).

Linear regression analysis was performed in order to investigate any relationship between the lenticule thickness or the ablation depth (independent variables) and the postoperative CD values (dependent variable) (Figure 3). In SMILE group, with the exception of CD of central corneal layers, the CD of the anterior 120 *μ*m, posterior 60 *μ*m, and total showed no statistical correlation with the lenticule thickness. Specifically, there was a weak negative association between the postoperative CD of the anterior 120 *μ*m and the thickness of the extracted lenticule (*P* = 0.108, coefficient of determination *R*^2^ = 0.046, ANOVA). The CD of the central corneal layers presented a statically significant inverse association with the thickness of the lenticule (*P* = 0.037, *R*^2^ = 0.0774). The CD of the posterior 60 *μ*m showed a weak negative association with the lenticule thickness (*P* = 0.271, *R*^2^ = 0.0214). The total postoperative CD (all corneal layers) at 0–6 mm annulus displayed a weak inverse association with the lenticule thickness, without reaching statistical significance (*P* = 0.079, *R*^2^ = 0.0532). In FS-LASIK group, the postoperative CD of the anterior 120 *μ*m at 0–6 mm annulus showed a weak positive association with the ablation depth (*P* = 0.857, *R*^2^ = 0.0007). The regression model indicated a weak negative association between the postoperative CD of the central corneal layers (*P* = 0.665, *R*^2^ = 0.0034), the posterior 60 *μ*m (*P* = 0.410, *R*^2^ = 0.0128), and all corneal layers (*P* = 0.731, *R*^2^ = 0.0015) with the ablation depth (Figure 3).

### 3.3. Visual and Refractive Outcomes

Regarding the CDVA in SMILE group, there were no significant differences observed between preoperative and postoperative values. Out of the 58 eyes that underwent SMILE, 9 eyes were primarily undercorrected. Preoperatively, the mean spherical equivalent (SE) was −5.76 ± 1.80 (range −9.50 to −2.0) and the CDVA was 1.04 ± 0.2 (range 0.63 to 1.60). Postoperatively, the mean SE was −0.44 ± 0.64 (range −2.13 to 0.50) and the CDVA was 1.04 ± 0.16 (range 0.63 to 1.40). There were no significant differences of CDVA in SMILE group before and 3 months after surgery (*P* = 0.509). From the 58 eyes, 1 eye lost 2 lines, 9 lost 1 line, 37 remained unchanged, 9 gained 1 line, and 2 gained 2 lines. In FS-LASIK group, 6 out of 58 eyes were primarily undercorrected. The mean SE was preoperatively −5.03 ± 2.32 (range −9.88 to −0.50) and postoperatively −0.32 ± 0.39 (range −1.63 to 0.50). The preoperative CDVA was 1.03 ± 0.13 (range 0.8 to 1.40) and postoperatively changed to 1.05 ± 0.13 (range 0.80 to 1.40). Similarly to SMILE group, there were no significant differences in FS-LASIK group before and at three months after surgery (*P* = 0.163) (Table 4). After FS-LASIK, 7 eyes lost 1 line, 40 remained unchanged, 9 gained 1 line, and 2 gained 2 lines. Comparing the postoperative CDVA between the two groups, we found no significant differences (*P* = 0.517) (Table 5).

## 4. Discussion

The Scheimpflug corneal densitometry is a fast and noninvasive method for assessing the backscatter profile of the entire cornea (up to a 12 mm zone), characterized by accuracy, reproducibility, and repeatability [10]. Other methods of evaluating corneal transparency are optical coherence tomography (OCT) [22] and in vivo confocal microscopy (IVCM) [23].

Light-backscattering analysis with OCT seems to be a repeatable method [22]. The analysis, however, is limited to a single cross-sectional image. In the publication from Wang et al., light-backscattering was evaluated on a single-line scan, with 1.13 mm length. In our study, CD data were obtained from a series of 25 images (1003 × 520 pixels) over different meridians.

IVCM is used in order to examine corneal endothelium, to assess cellular morphology, to evaluate cellular responses and nerve regeneration, and to monitor inflammatory corneal processes, such as acanthamoeba and fungal keratitis. IVCM is also a useful tool for quantifying corneal backscattered light, enabling therefore an accurate assessment of the stroma reaction, activation of keratocytes, and haze grading after corneal refractive surgery [9, 23, 24]. Despite the fact that IVCM provides a high degree of magnification and resolution, it remains an invasive method which evaluates backscatter profile of a very small area (0.14% of the total cornea) [10, 23].

The Scheimpflug corneal densitometry and IVCM utilize different measurement principles. The Scheimpflug analysis, due to the noncontact nature of the method, results in higher reflection at the interfaces between layers with different indices of refraction, that is, air–cornea interface, epithelium–anterior stroma, and posterior stroma–endothelium [10]. Furthermore, Scheimpflug systems illuminate the cornea perpendicularly and analyze the corneal cross-section from an angle of ±45°. By IVCM, the reflection at the air–cornea interface is eliminated by the use of water-immersion front lens. Moreover, IVCM illuminates and acquires images of the cornea perpendicularly, which results in higher amounts of specularly reflected light than with Scheimpflug [10].

The transitory enhanced visibility of the interface after SMILE and FS-LASIK procedures is mainly associated with the wound healing and inflammatory response. During a SMILE procedure, the photodisruption, induced by the femtosecond laser (solid-state Nd:Glass laser, wavelength 1043 nm, pulse duration 220–580 femtoseconds, laser pulse repetition rate 500 kHz, and spot size ~1 *μ*m) for the lenticule creation, produces a plasma, shockwave, and cavitation bubble. The low-energy profile, the reduced pulse duration, and high laser firing speed, provided by the femtosecond laser platform, result in reduced cavitation bubble size (spot size) and breakdown threshold of plasma formation, therefore minimizing collateral damage and tissue inflammation. Moreover, the 1043 nm wavelength of light pulses is not absorbed by corneal tissue and the thermal effect on the cornea is minimal [25, 26].

During FS-LASIK, besides the photodisruptive effect of the femtosecond laser for the flap creation, the cornea is additionally burdened by the effect of the excimer laser (argon fluoride laser, wavelength 193 nm, frequency 250 Hz, and spot size 0.7 mm). During the ablative photodecomposition induced by the excimer laser, high-energy photons break the organic molecular bonds within corneal tissue [7]. Contrary to femtosecond laser, the 193 nm wavelength of light pulses is absorbed by corneal tissue, causing further thermal along with secondary radiation damage [27].

The tissue trauma following the excimer laser photoablation releases various cytokines and chemokines that modulate the corneal wound healing process [7, 28]. Riau et al. have shown that, in cases of all-femtosecond laser-assisted procedures such as the refractive lenticule extraction, there was little or no expression of early inflammatory markers in the central stroma and, moreover, their number remained stable regardless of the power of the correction. On the contrary, after FS-LASIK, the expression of early inflammatory markers increased significantly when high-power corrections were performed and cornea reflectivity, as examined with IVCM, showed more intense and abundant light-scattering particles as a result of the photoablation process [7]. Dong et al. showed that SMILE may stimulate less keratocyte proliferation and tissue inflammation compared to FS-LASIK. Moreover, greater keratocyte apoptosis was induced after FS-LASIK, probably due to flap lifting and greater contact of the bare stroma with cytokines induced by the epithelial trauma. Another major experimental study was recently presented by Liu et al. investigating the postoperative wound healing response after hyperopic SMILE, hyperopic SMILE without lenticule extraction, and hyperopic FS-LASIK. The authors concluded that hyperopic SMILE induced less postoperative wound healing response and stromal interface reaction compared to hyperopic FS-LASIK, especially in higher refractive correction. Moreover, the keratocyte response was upregulated after hyperopic SMILE when compared to hyperopic SMILE without lenticule extraction, suggesting that the surgical manipulation, rather than the laser, might induce cellular stress in the surrounding stromal tissue [8].

A limitation of our study would be that it does not evaluate the short-term, transient effects over the first postoperative weeks for example, 1 week and 1 month. However, the results of CD measurements during that period would have possibly been affected by disturbances of the tear film, which are common during that period. Moreover, the purpose of this study was not to investigate the short-term effect of SMILE and FS-LASIK on corneal clarity but to objectively assess if this effect (“transient haze”) persists in the midterm for example, 3 months after surgery.

Our results for total CD showed for SMILE group similar values preoperatively and 3 months postoperatively (Table 2**)**. Moreover, both SMILE and FS-LASIK groups showed a similar CD status at 3 months after surgery and a weak inverse relationship to the amount of the removed tissue (Table 5). Postoperative corneal clarity after SMILE might be limited due to the intensity and duration of interface remodeling and could be associated with the femtosecond laser specifications, such as the repetition rate [25, 26, 29], with the remained interface debris (cellular constituents) inside the SMILE pocket, with the scanning pattern of the femtosecond laser [30] or with the surgeon factor (more surgical maneuvers could result in greater tissue trauma and inflammatory response). Each one or a combination of these factors could result in higher corneal opacification and slower visual recovery. The VisuMax femtosecond laser used for our SMILE cases had a repetition rate at 500 kHz with the lamellar cuts being performed in a spiral in/out scanning pattern direction. Moreover, the stromal pocket was thoroughly flushed with BSS in order to remove part of the remained interface debris (cellular constituents). Finally, the procedures were performed by an experienced surgeon and therefore the surgical maneuvers were minimized, resulting in less tissue trauma and inflammatory response. However, even in our cases, the CD of the anterior 120 *μ*m was significantly increased 3 months after surgery. Although such an increase would be considered subclinical and had no impact on visual acuity, it may suggest a less intense but yet ongoing stromal remodeling at the level of the interface, for example, approximately 120 *μ*m.

Another interesting aspect of our results is the increased transparency of the posterior stroma in both groups (statistically significant reduction of CD). Although in both groups the CD values of the posterior stroma were weakly related with the extracted or photoablated corneal stroma (Figure 3), it could be possible that the increase in posterior stroma transparency is associated with the quality of the acquired images from this layer (the reduced overlying stroma would result in less remaining backscatter particles, such as less keratocytes and collagen fibrils, and might have enabled a more accurate evaluation of the backscattered light from the posterior stroma).

Finally, the regression analysis showed that the corneal clarity in the midterm is not correlated with the amount of the extracted or ablated stromal tissue. However, the statistically significant inverse correlation of the central CD with the lenticule thickness in SMILE group may be associated with the higher volume of the extracted stromal tissue (compared to the volume of the ablated stromal tissue in FS-LASIK), which would result in greater reduction of keratocyte density and therefore less amount of backscattered light from the remaining central corneal layers.

## 5. Conclusions

Our study showed an equal corneal clarity after SMILE and FS-LASIK at 3 months after surgery, as evaluated with the Scheimpflug corneal densitometry. The association between the postoperative total CD and the lenticule thickness or the ablation depth was insignificant. Visual outcomes showed no significant differences of CDVA preoperatively and 3 months postoperatively within its group and between them. We speculate that the transitory enhanced visibility of the interface which might be observed after SMILE could be associated with the amount of debris inside the stromal pocket and the subsequent inflammatory response in corneal stroma, as well as the tissue remodeling. This in turn might be associated with the degree of mechanical manipulation and/or collateral damage caused by laser-tissue interaction. From this point of view, a thorough irrigation of the stromal pocket might result in removal of the apoptotic and necrotic keratocytes as well as denatured collagen from the pocket and the interface edges, leading to less inflammation, faster stromal remodeling, and better postoperative corneal transparency.

## Figures and Tables

**Table 1:** SMILE versus FS-LASIK—demographics, preoperative, and surgery data.

Groups	SMILE	FS-LASIK	*P*
Patients	33	33	—
Total eyes (right/left)	30/28	28/30	—
Gender (M/F)	15/18	13/20	—
Mean age (y)	38 ± 10 (23 to 56)	37 ± 10 (23 to 52)	0.086
Mean SE (D)	−5.76 ± 1.80 (−9.5 to −2.0)	−5.03 ± 2.32 (−9.88 to −0.5)	0.108
Mean cylinder (D)	−0.99 ± 0.90 (−4.25 to 0)	−0.95 ± 0.94 (−5.0 to 0)	0.982
White to white (mm)	12.2 ± 0.3 (11.4 to 12.9)	12.2 ± 0.3 (11.8 to 12.8)	0.340
*Surgery data*			
Mean SE of the correction (D)	−5.51 ± 1.86 (−9.50 to −1.75)	−4.80 ± 2.4 (−10.0 to −0.8)	0.136
Mean cylinder of the correction (D)	−0.86 ± 0.94 (−4.0 to 0)	−0.90 ± 0.90 (−4.5 to 0)	0.773
Mean flap/cap thickness (*μ*m)	119 ± 2 (110 to 120)	113 ± 6 (100 to 120)	<0.001
Mean flap/cap diameter (mm)	7.81 ± 0.07 (7.7 to 8.0)	8.35 ± 0.10 (8.0 to 8.5)	<0.001
Mean lenticule thickness/ablation depth (*μ*m)	116 ± 28 (48 to 172)	99 ± 38 (22 to 165)	0.017
Mean lenticule/ablation diameter (mm)	6.72 ± 0.19 (6.3 to 7.0)	6.26 ± 0.21 (6 to 6.50)	<0.001

SE: spherical equivalent; D: diopters.

**Table 2:** SMILE—preoperative versus 3-month postoperative corneal densitometry.

SMILE				
		0–2 mm	2–6 mm	Total (0–6 mm annulus)
Anterior (120 *μ*m)	Preop	24.8 ± 1.6 (19.6 to 27.1)	22.4 ± 1.7 (16.1 to 25.7)	23.6 ± 1.6 (17.9 to 26.4)
	3 months postop	25.1 ± 1.8 (20.8 to 28.9)	23.0 ± 1.8 (16.7 to 26.3)	24.0 ± 1.8 (18.8 to 27.6)
	*P* value	0.105	<0.001	0.003

Central layer	Preop	15.4 ± 1.0 (12.3 to 17.2)	14.1 ± 1.1 (10.2 to 16.6)	14.8 ± 1.0 (11.3 to 16.9)
	3 months postop	15.6 ± 1.0 (13.2 to 17.4)	14.2 ± 1.0 (10.5 to 15.8)	14.9 ± 1.0 (11.9 to 16.5)
	*P* value	0.015	0.317	0.078

Posterior (60 *μ*m)	Preop	13.0 ± 1.0 (9.7 to 15.9)	12.2 ± 1.1 (8.7 to 15.3)	12.6 ± 1.1 (9.2 to 15.6)
	3 months postop	12.6 ± 0.9 (10.9 to 14.9)	12.0 ± 1.0 (9.3 to 13.8)	12.3 ± 0.9 (10.1 to 14.3)
	*P* value	0.001	0.264	0.012

Total (all layers)	Preop	17.8 ± 1.1 (13.9 to 20.0)	16.2 ± 1.2 (11.6 to 19.0)	17.0 ± 1.2 (12.8 to 19.5)
	3 months postop	17.8 ± 1.1 (15.0 to 19.8)	16.4 ± 1.2 (12.2 to 18.4)	17.1 ± 1.1 (13.6 to 19.0)
	*P* value	0.877	0.073	0.259

**Table 3:** FS-LASIK—preoperative versus 3-month postoperative corneal densitometry.

FS-LASIK				
		0–2 mm	2–6 mm	Total (0–6 mm annulus)
Anterior (120 *μ*m)	Preop	26.0 ± 1.7 (23.5 to 30.6)	23.5 ± 1.7 (21.1 to 29.0)	24.8 ± 1.7 (22.4 to 29.8)
	3 months postop	25.8 ± 1.7 (21.7 to 29.0)	23.6 ± 1.7 (20.0 to 27.8)	24.7 ± 1.6 (20.9 to 28.4)
	*P* value	0.236	0.465	0.815

Central layer	Preop	15.9 ± 0.9 (14.5 to 17.7)	14.7 ± 1.0 (13.0 to 16.9)	15.3 ± 0.9 (13.8 to 17.1)
	3 months postop	15.7 ± 0.9 (13.9 to 18.0)	14.6 ± 1.1 (12.8 to 17.4)	15.2 ± 1.0 (13.5 to 17.7)
	*P* value	0.020	0.222	0.059

Posterior (60 *μ*m)	Preop	13.3 ± 0.9 (11.1 to 15.1)	12.6 ± 1.0 (10.7 to 15.3)	12.9 ± 0.9 (10.9 to 15.1)
	3 months postop	12.7 ± 0.9 (10.9 to 15.0)	12.4 ± 1.0 (10.1 to 15.3)	12.5 ± 0.9 (10.5 to 15.2)
	*P* value	<0.001	0.045	0.001

Total (all layers)	Preop	18.4 ± 1.0 (16.6 to 20.4)	16.9 ± 1.1 (15.3 to 19.7)	17.7 ± 1.1 (16.0 to 20.1)
	3 months postop	18.0 ± 1.0 (15.9 to 20.2)	16.8 ± 1.2 (14.6 to 19.4)	17.4 ± 1.1 (15.4 to 19.8)
	*P* value	0.002	0.417	0.033

**Table 4:** Comparison of preoperative and 3-month postoperative refractive data and CDVA: SMILE versus FS-LASIK.

	Preoperatively	3 months postoperatively	*P* value
*SMILE*			
Mean sphere (D)	−5.26 ± 1.88 (−9.50 to −1.75)	−0.17 ± 0.41 (−1.50 to 0.50)	
Mean cylinder (D)	−0.99 ± 0.90 (−4.25 to 0)	−0.31 ± 0.37 (−1.25 to 0)	
Mean SE (D)	−5.76 ± 1.80 (−9.5 to −2.0)	−0.44 ± 0.64 (−2.13 to 0.5)	
CDVA	1.04 ± 0.20 (0.63 to 1.60)	1.04 ± 0.16 (0.63 to 1.40)	0.509

*FS-LASIK*			
Mean sphere (D)	−4.56 ± 2.36 (−9.50 to −0.25)	−0.22 ± 0.37 (−1.50 to 0.50)	
Mean cylinder (D)	−0.95 ± 0.94 (−5.0 to 0)	−0.20 ± 0.24 (−1.0 to 0)	
Mean SE (D)	−5.03 ± 2.32 (−9.88 to −0.50)	−0.32 ± 0.39 (−1.63 to 0.5)	
CDVA	1.03 ± 0.13 (0.80 to 1.40)	1.05 ± 0.13 (0.80 to 1.40)	0.163

CDVA: corrected distance visual acuity (decimal values).

**Table 5:** Comparison of preoperative and 3-month postoperative total corneal densitometry (all corneal layers) for the different annuli and CDVA: SMILE versus FS-LASIK.

	Preoperatively	3 months postoperatively
*0–2 mm annulus (all corneal layers)*		
SMILE	17.8 ± 1.1 (13.9 to 20.0)	17.8 ± 1.1 (15.0 to 19.8)
FS-LASIK	18.4 ± 1.0 (16.6 to 20.4)	18.0 ± 1.0 (15.9 to 20.2)
*P* value	0.11	0.569

*2–6 mm annulus (all corneal layers)*		
SMILE	16.2 ± 1.2 (11.6 to 19.0)	16.4 ± 1.2 (12.2 to 18.4)
FS-LASIK	16.9 ± 1.1 (15.3 to 19.7)	16.8 ± 1.2 (14.6 to 19.4)
*P* value	0.05	0.055

*0–6 mm annulus (all corneal layers)*		
SMILE	17.0 ± 1.2 (12.8 to 19.5)	17.1 ± 1.1 (13.6 to 19.0)
FS-LASIK	17.7 ± 1.1 (16.0 to 20.1)	17.4 ± 1.1 (15.4 to 19.8)
*P* value	0.007	0.686

*CDVA*		
SMILE	1.04 ± 0.20 (0.63 to 1.60)	1.04 ± 0.16 (0.63 to 1.40)
FS-LASIK	1.03 ± 0.13 (0.80 to 1.40)	1.05 ± 0.13 (0.80 to 1.40)
*P* value	0.805	0.517
